# Translation Regulation by eIF2α Phosphorylation and mTORC1 Signaling Pathways in Non-Communicable Diseases (NCDs)

**DOI:** 10.3390/ijms21155301

**Published:** 2020-07-26

**Authors:** Tiffany J. Rios-Fuller, Melanie Mahe, Beth Walters, Dounia Abbadi, Sandra Pérez-Baos, Abhilash Gadi, John J. Andrews, Olga Katsara, C. Theresa Vincent, Robert J. Schneider

**Affiliations:** 1 Department of Microbiology, NYU School of Medicine, New York, NY 10016, USA; Tiffany.Rios@nyulangone.org (T.J.R.-F.); Melanie.Mahe@nyulangone.org (M.M.); beth.walters@nyulangone.org (B.W.); Dounia.Abbadi@nyulangone.org (D.A.); Sandra.Perezbaos@nyulangone.org (S.P.-B.); Abhilash.Gadi@nyulangone.org (A.G.); john.andrews@nyulangone.org (J.J.A.); Olga.Katsara@nyulangone.org (O.K.); Theresa.Vincent@nyulangone.org (C.T.V.); 2 Department of Immunology, Genetics and Pathology, Uppsala University, Rudbeck Laboratory, 751 85 Uppsala, Sweden

**Keywords:** translation, non-communicable diseases, eIF2 stress, mTOR signaling

## Abstract

Non-communicable diseases (NCDs) are medical conditions that, by definition, are non-infectious and non-transmissible among people. Much of current NCDs are generally due to genetic, behavioral, and metabolic risk factors that often include excessive alcohol consumption, smoking, obesity, and untreated elevated blood pressure, and share many common signal transduction pathways. Alterations in cell and physiological signaling and transcriptional control pathways have been well studied in several human NCDs, but these same pathways also regulate expression and function of the protein synthetic machinery and mRNA translation which have been less well investigated. Alterations in expression of specific translation factors, and disruption of canonical mRNA translational regulation, both contribute to the pathology of many NCDs. The two most common pathological alterations that contribute to NCDs discussed in this review will be the regulation of eukaryotic initiation factor 2 (eIF2) by the integrated stress response (ISR) and the mammalian target of rapamycin complex 1 (mTORC1) pathways. Both pathways integrally connect mRNA translation activity to external and internal physiological stimuli. Here, we review the role of ISR control of eIF2 activity and mTORC1 control of cap-mediated mRNA translation in some common NCDs, including Alzheimer’s disease, Parkinson’s disease, stroke, diabetes mellitus, liver cirrhosis, chronic obstructive pulmonary disease (COPD), and cardiac diseases. Our goal is to provide insights that further the understanding as to the important role of translational regulation in the pathogenesis of these diseases.

## 1. Introduction

An understanding of complex NCDs provides mechanistic insights and often yields potential therapeutic targets. While the translation machinery has not traditionally been thought of as druggable target, the past decade has witnessed a rapid development of many specific therapeutics that target the translation machinery, its upstream regulatory protein kinases (particularly mTOR), and specific translation factors for the treatment of a variety of human diseases, with a strong focus on oncology. Moreover, the expression levels and activities of certain translation factors and regulatory proteins have become reliable biomarkers for measuring the efficacy of targeted therapies in both the prevention and treatment of NCDs. The original classification of diseases was first established by Jacques Bertillon in 1893 and relied on the affected anatomical site rather than specific pathology or characteristics [1]. Unlike most infectious diseases, which are complex, the additional complexity of NCDs is introduced by the long latency periods involved in their development, the association of multiple genetic and risk factors that underlie the development of NCDs, that often involve several co-morbidities, and the prolonged course of disease [2].

According to the World Health Organization (WHO), NCDs are the major cause of mortality and morbidity, contributing to 41 million (71%) deaths worldwide in 2016 [3]. The four most prevalent NCDs worldwide are cardiovascular disease, cancer, chronic respiratory disease, and diabetes [4]. Many NCDs have common underlying causes, including excessive alcohol use, smoking, obesity, and untreated hypertension (elevated blood pressure), with effects likely sharing common signaling transduction pathways [5,6,7,8,9]. Nutritional effects, which can contribute to the development of some NCDs, can alter levels of growth factors, hormones, and cellular receptors that regulate signaling pathways that control both transcriptional and translational gene expression [10]. These alterations regulating translation initiation can directly control gene expression levels, with neurological, endocrine, pulmonary, and cardiovascular functions commonly disrupted [11,12,13,14,15].

**Figure 1:**
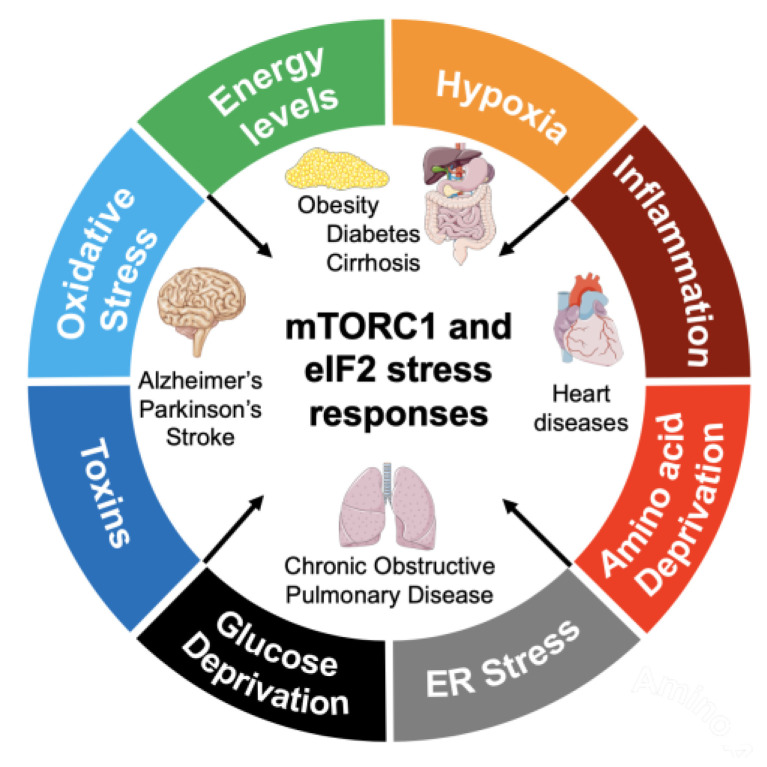
Pathological regulation of mTORC1 and eIF2 stress response in non-communicable diseases. Many cellular stress conditions in different tissues and organs involve activation of the ISR with eIF2α phosphorylation and dysregulation of the mTORC1 signaling pathway. Particularly affected are the brain, liver, pancreas, lungs, and heart, which contribute to chronic diseases such as Alzheimer’s, Parkinson’s, stroke, diabetes mellitus, liver cirrhosis, chronic obstructive pulmonary disease, and heart diseases.

In this review, we will briefly provide an overview of the different processes of translation initiation and its regulation. We will then focus on two major mechanisms of translational regulation that are often involved in the pathology of NCDs: the eIF2 ISR pathway, which includes endoplasmic reticulum (ER) stress and unfolded protein response (URP) [16], and the mTORC1 signaling pathway. While there are many NCDs in which the eIF2/ISR and mTORC1 pathways are involved, we will focus on Alzheimer’s disease, Parkinson’s disease, stroke, diabetes mellitus, liver cirrhosis, chronic obstructive pulmonary disease, and heart diseases (Figure 1). In all these cases there is a clear contribution from the eIF2/ISR and mTORC1 pathways to disease pathogenesis and potential therapeutic opportunities. 

### 1.1. A Brief. Overview of mRNA Translation Initiation in Eukaryotes

**Figure 2:**
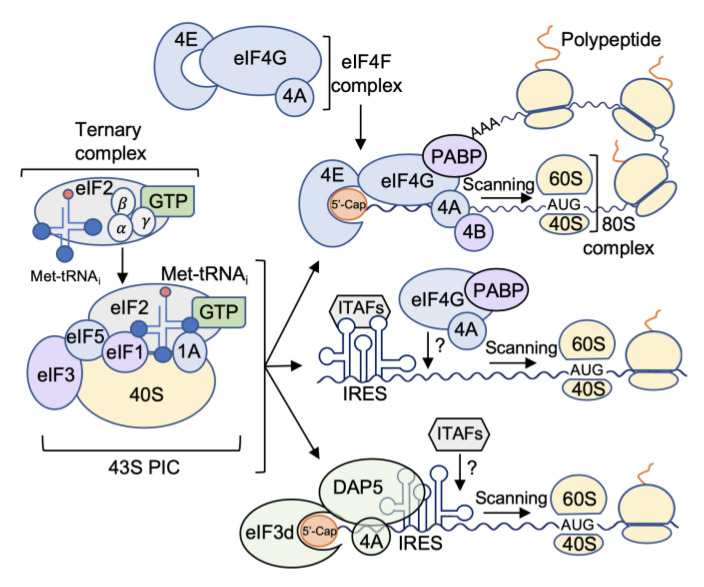
mRNA translation initiation in eukaryotes. Translation initiation begins with the formation of the ternary complex, comprised of eIF2, GTP, and the initiator Met- tRNA_i_. The ternary complex is recruited to the 40S ribosome subunit along with eIF1, eIF1A, eIF3, and eIF5, to form the 43S preinitiation complex (43S PIC). Briefly, during canonical cap-dependent translation, the mRNA is bound by the eIF4F complex comprised of eIF4E (the cap-binding protein), eIF4G (scaffolding protein), and eIF4A (an ATP-dependent RNA helicase), which is then recruited to the 43S PIC, along with the poly(A)-binding protein (PABP) and eIF4B. During cap-independent or internal ribosome entry, known as (IRES)-mediated translation, some IRES trans-acting factors (ITAFs) can function as RNA chaperones to change or stabilize secondary structures of the IRES allowing ribosome binding to the IRES, or as adaptor proteins to interact with the ribosome or translation initiation factors. Finally, during alternate cap-dependent translation, DAP5 together with the cap-binding activity of eIF3d carries out cap-dependent translation through a mTORC1/eIF4E cap-independent mechanism. Once the 43S PIC is bound to the mRNA, it scans in a 5′ to 3′ direction until it recognizes an optimal start codon (AUG is shown). Recognition of the start codon triggers the release of eIF1 and hydrolysis of eIF2-GTP to its GDP-bound state (not shown). eIF1A stimulates the binding of the 60S ribosomal subunit to form an elongation-competent 80S ribosome. The 80S complex, which has peptidyltransferase activity that catalyzes polypeptide synthesis, enters the elongation phase of translation.

Translation initiation in eukaryotes is a highly regulated process with the majority (>95%) of initiation events occurring through a cap-dependent mechanism [17]. In brief, during cap (m7G)-dependent initiation, eukaryotic initiation factor (eIF) 4E binds the 5′-cap, the scaffolding factor eIF4G, the ATP-dependent RNA helicase eIF4A, the poly(A) binding protein (PABP), and the multi-protein factor eIF3. This complex recruits the 43S preinitiation complex (43S PIC) consisting of the 40S ribosomal small subunit and the ternary complex (TC) formed by eIF2, GTP, and the initiator methionyl-transfer RNA (Met-tRNA_i_), as well as a number of additional factors (Figure 2) [18]. Once loaded onto the mRNA, the PIC scans the 5′ untranslated region (5′-UTR) of the mRNA in a 5′ to 3′ direction until recognition of the optimal start codon, which is often but not always an AUG [19]. Upon recognition of a start codon, eIF1 is partially displaced to enable the Met-tRNA_i_ to establish a codon-anticodon pairing with the mRNA, the 60S ribosome subunit joins, the GTP on eIF2 is hydrolyzed concurrent the start of protein synthesis and must undergo GDP to GTP exchange to allow subsequent rounds of initiation [20]. Further, eIF5 and eIF5B facilitate the dissociation of certain initiation factors, and eIF1A remains bound to the 40S ribosome, stimulating the binding of the 60S ribosomal subunit to form a translationally active and elongation-competent 80S ribosome [21]. Finally, the 80S complex, which has peptidyltransferase activity that catalyzes polypeptide synthesis, enters the elongation phase of translation [18]. 

### 1.2. Overview of Regulation of Translation Initiation by the eIF2/ISR and mTORC1 Pathways

#### 1.2.1. eIF2/ISR Regulation of Translation Initiation

Translation initiation is downregulated during the ISR by a mechanism involving the phosphorylation of the eIF2 α-subunit at Ser-51 [22,23]. As mentioned, during translation initiation, eIF2 forms a ternary complex with Met-tRNA_i_ and GTP, which binds the 40S subunit to form the 43S PIC (Figure 2) [24]. Phosphorylation of eIF2α at Ser-51 dramatically increases the binding affinity of eIF2 for its guanine nucleotide exchange factor (GEF) known as eIF2B, resulting in sequestration that blocks eIF2-GDP to eIF2-GTP exchange activity and consequently inhibits translation [25,26]. Different stress conditions provoke eIF2α phosphorylation by any of four related protein kinases: the heme-regulated inhibitor (HRI) in red blood cells, the protein kinase RNA-activated (PKR) that responds to viral infection, the PKR-like endoplasmic reticulum (ER) kinase (PERK) that senses ER stress in the ISR/UPR pathway, and the general control non-depressible 2 kinase (GCN2) that responds to a deficit in levels of certain amino acids [27]. The HRI kinase (or EIF2AK1) has two main roles during development; it ensures a balanced synthesis of globin and heme and promotes the survival of erythroid precursors during iron deficiency [28]. It was initially thought that HRI expression is limited to erythrocytes, but recent studies have demonstrated that it is also present in the liver and macrophages [29,30]. PKR (or EIF2AK2) was initially known as a kinase that is activated by double-stranded RNA (dsRNA) typically activated during viral infection, and blocks the translation of viral mRNAs [31]. However, PKR can become activated in response to other signals such as oxidative and ER stress, or cytokine and growth factor signaling [32,33,34]. PERK (EIF2AK3) is mainly activated by the accumulation of misfolded proteins in the ER, termed ER stress [35]. The cellular response to ER stress involves increased expression of ER chaperones and folding enzymes to refold misfolded proteins, a process called “unfolded protein response” (UPR), and degradation of those proteins that are terminally misfolded, which can activate the process of endoplasmic-reticulum-associated protein degradation (ERAD) [36]. Aiming to restore ER homeostasis, phosphorylation of eIF2α by PERK inhibits the synthesis of new polypeptides, thus reducing the access of nascent polypeptides to the ER lumen [37]. GCN2 (EIF2AK4) is the major regulator of gene expression in response to amino acid limitation and is critical for maintaining metabolic homeostasis during glucose deprivation [38,39]. Importantly, brief ISR is an adaptive and pro-survival response, aiming to relieve stress, regulate ribosome biogenesis, and restore homeostasis, while prolonged ISR can induce cell death by activating apoptosis [40,41]. Translation of the mammalian activating transcription factor 4 (ATF4) during ER stress has been well-reviewed by others [42]. ATF4 translation involves two short upstream open reading frames (uORFs): uORF1 located in the 5′-UTR of the ATF4 mRNA and uORF2 that overlaps out-of-frame with the ATF4 coding region [43,44]. When GTP-eIF2 levels in non-stressed cells are high, scanning of 40S ribosomes downstream from uORF1 will reinitiate translation at inhibitory uORF2, resulting in no ATF4 expression. However, when GTP-eIF2 levels are low, due to elevated levels of eIF2α phosphorylation in stressed cells, there is a delay in re-initiation that allows the scanning ribosomes to bypass the inhibitory uORF2, leading to increased translation of the ATF4 coding region [43]. Activation of ATF4 protects cells against oxidative and ER stress and ensures amino acid availability for proteins [45].

#### 1.2.2. mTORC1 Regulation of Translation Initiation

In mammalian cells, phosphoinositide 3-kinase (PI3K) is activated by several upstream activators, in particular receptor-coupled tyrosine kinases (RTK) [46]. When an RTK is activated, PI3K complex forms and phosphorylates the inositol ring of phosphatidylinositol-4-5-biphosphate (PIP_2_) to generate phosphatidylinositol-3,4,5-triphosphate (PIP_3_), a secondary messenger that recruits cytoplasmic proteins to the endo- or plasma membrane [47]. Upon PIP_3_ formation, protein kinase B-serine/threonine kinase (AKT) and its upstream activating kinase, phosphoinositide-dependent kinase-1 (PDK-1), translocate from the cytoplasm to the plasma membrane [47]. Full activation of AKT requires phosphorylation of two residues: Thr308 by the constitutively active PDK-1, and Ser473 by the mammalian target of the rapamycin complex 2 (mTORC2) [48]. AKT was the first kinase shown to directly phosphorylate the tuberous sclerosis proteins 1 and 2, also known as TSC1 (hamartin) and TSC2 (tuberin) in response to growth factors. The TSC1–TSC2 complex plays a central role in the inhibition of mTOR, a serine/threonine-protein kinase [49,50]. mTOR functions in two multi-subunit protein complexes, mTORC1, and mTORC2, each with its own distinct subunit composition and substrate selectivity [51]. The small GTPase Rheb (Ras homolog enriched in brain) is a direct target of TSC2, a GTPase-activating protein (GAP) stabilized by TSC1, with the activation of the TSC1/TSC2 complex inhibiting mTORC1 by stimulating the conversion of GTP-Rheb to GDP-Rheb [51]. Phosphorylation of TSC2 by AKT causes dissociation from TSC1, preventing the complex formation and allowing GTP-Rheb to activate mTORC1 [52]. The nutrient-sensitive mTORC1 complex regulates ribosome biogenesis by promoting the translation of mRNAs encoding all of the cytoplasmic ribosomal proteins, and by stimulating transcription of ribosomal RNAs (rRNAs) [53,54,55]. mTORC1 stimulates translation and cell proliferation through the phosphorylation of two main targets of ribosome biogenesis, the p70 ribosomal protein S6 kinase (p70S6K), which induces translation by ribosomal protein S6 (a component of the 40S ribosome), and the 4E-BPs (the eIF4E binding proteins (1, 2, and 3), regulating canonical translation initiation [56,57,58]. mTOR-mediated S6K has an extensive effect on ribosome biogenesis, providing the cell with nucleolar factors required for rRNA synthesis and post-transcriptional modifications [59]. Over 75% of ribosome biogenesis factors in mouse liver are controlled by S6K, showing the critical role of the mTORC1/S6K1/rpS6 axis for ribosome biogenesis program and as a potential therapeutic target [59]. Further, when 4E-BPs are phosphorylated they become inactive and undergo a structural change that inhibits their binding to eIF4E, allowing eIF4E to interact with eIF4G. However, when 4E-PBs are dephosphorylated, they are active and sequester eIF4E, inhibiting binding to eIF4G and the 5′-cap, which downregulates canonical translation initiation [60]. In addition, cyclin-dependent kinase 1 (CDK1) acts as a general activator of translation allowing direct adaptation of protein synthesis [61,62], and is able to phosphorylate the 4E-BPs under conditions when mTOR signaling is decreased by activating mitotic cap-dependent mRNA translation [63]. It is particularly notable that the mTORC1 pathway has been shown to be hyperactivated during mitosis, despite decreased global protein synthesis and decreased activity of mTORC1 upstream activators, with abnormal cell proliferation and protein synthesis [64]. This implies that there are non-canonical mechanisms for the maintenance of cap-dependent mRNA translation that does not involve mTORC1/eIF4E-mediated translation and may be at play during the development and progression of NCDs. Studies need to be carried out that avoid issues related to cell-cycle synchronization, which may be inducing a decrease in global protein synthesis unrelated to translation signaling [65,66,67]. 

#### 1.2.3. Alternate Mechanisms of mRNA Translation Initiation

Some cellular mRNAs are able to initiate translation through either a cap using the conventional PIC or through a cap-independent mechanism using internal ribosome entry site (IRES) elements [68]. An IRES is thought to contain one or more highly structured elements possibly with single-stranded density in the 5′-UTR of the mRNA that may serve as a landing site for eIF4G family members, eIF3 or even the 40S ribosome subunit itself, thereby bypassing a requirement for eIF4E in initiation [69,70]. IRES-mediated translation is fundamentally important to promote cell survival and other responses during cell stress that downregulates mTORC1 activity, eIF4E availability, and thereby allows translation of certain mRNAs that adaptively respond to cell stress conditions. While many viral IRESs have been well characterized and classified into distinct functional types based on their sequence, secondary structures, and a requirement for canonical and non-canonical translation factors, relatively little is understood regarding how cellular IRESs function and are recognized by the translation machinery [71]. There are few structural or sequence similarities observed between viral and cellular IRESs, and among cellular IRESs [72]. A number of proteins have been identified which are capable of interacting with IRESs are called IRES *trans*-acting factors (ITAFs) [70,73]. Some ITAFs can function as RNA chaperones to change or stabilize secondary structures of the IRES allowing ribosome binding to the IRES, or as adaptor proteins to interact with the ribosome or translation initiation factors (Figure 2) [69,74]. Importantly, IRESs are capable of binding only a subset of initiation factors, which in turn can recruit the 40S subunit internally to the mRNA [75].

In addition to mTORC1/eIF4E-directed translation initiation of capped mRNAs, approximately 20% of capped mRNAs were recently shown to also utilize an alternate PIC [75]. While it had long been thought that all cap-dependent mRNA translation requires eIF4E to direct the assembly of a PIC on mRNA, it was found that there is at least one additional mechanism that does not utilize eIF4E and does not require mTORC1 activity, and instead uses a member of the eIF4G family of proteins. The scaffolding protein eIF4G consists of three protein family members, eIF4GI (major form, highest expression, gene: *EIF4GI*), eIF4GII (minor form, lowest expression, gene: *EIF4G3*) and eIF4GIII (gene: *EIF4G2*), also known as DAP5 (NAT1 or p97) [76,77,78,79,80,81,82]. DAP5 is 65% homologous to the middle and C-terminus of canonical translation factor eIF4G1, but lacks the N-terminal region that interacts with cap-binding eIF4E, and PABP. Like eIF4GI, DAP5 can recruit eIF4A and eIF3, which bind to the 40S ribosome subunit and mediate non-canonical translation (Figure 2) [77,83]. DAP5 has been shown to be important for translation during stress responses by carrying out IRES-driven translation of certain cellular mRNAs such as proto-oncogene c-Myc [84], apoptosis regulator Bcl-2 [82], cyclin-dependent kinase 1 (CDK1) [82], apoptotic protease activating factor 1 (Apaf-1) [85], tumor protein p53 [86], X-linked inhibitor of apoptosis (XIAP) [87], and inhibitor of apoptosis protein 2 (c-IAP1/HIAP2) among others [88], in addition to its own mRNA, supporting its continuous translation and creating a positive autoregulatory loop [80,81,82,88,89,90]. DAP5 silencing results in a 20–30% reduction in overall protein synthesis which is far greater than IRES-mediated translation which is ~5%. Approximately 10–20% of mRNAs are solely dependent on DAP5 for their translation [76,91,92], as described below.

Studies have shown that DAP5 is important for the translation of certain capped mRNAs that do not seem to possess IRESs, under non-stress conditions. DAP5-dependent translation is required during cell proliferation where it is involved in the synthesis of cell cycle proteins, during translation of non-stressed cell survival mRNAs involved in mitosis, and translation of mRNAs that induce differentiation of human and mouse embryonic stem cells [82,93,94]. Indeed, it was recently discovered that DAP5 carries out an alternate mechanism of cap-dependent but eIF4E/mTORC1-independent mRNA translation, in addition to translating certain IRES containing mRNAs [76,77,78,79,80,81,82]. Translation of the majority of DAP5-dependent mRNAs requires the translation initiation factor eIF3d, a component of the multi-subunit factor eIF3, which has cap-binding activity [76,95]. DAP5/eIF3d-mediated cap-dependent mRNA translation has been shown to be involved in the translation of cell survival, proliferation, motility, DNA damage and repair response, and other mRNAs, most of which do not contain IRESs [76]. These findings suggest that DAP5/eIF3d is needed for both cap-independent and cap-dependent translation initiation mechanisms [76]. Interestingly, under ER stress, caspase-12 cleavage of DAP5/p97 produces a fragment known as p86, which enhances IRES-mediated translation of several mRNAs, leading to the reduction of apoptosis and allowing the UPR to establish survival mechanisms in response to cell stress [88,90]. Chronic ER stress is also characterized by an eIF3d-dependent mechanism of mRNA recruitment and limited translational recovery, critical for blocking ER overload while sustaining translation of mRNAs that encode stress-response factors, suggesting a role of DAP5/eIF3d translation during ER stress [80,96].

Finally, the mTOR mRNA itself may use two mechanisms for its translation, demonstrating that the mTOR mRNA utilizes both eIF4E-dependent under normal conditions and eIF4E-independent translation during cellular stress conditions, but which may not be DAP5/eIF3d-dependent [76,97]. In addition, the mTOR 5′-UTR forms a highly folded RNA scaffolding site, consisting of several stem-loops that can bind the 40S ribosomal subunit with high affinity, demonstrating a novel regulatory mechanism of mTOR gene expression and how it contributes in maintaining its biological functions [97]. 

It is therefore clear that certain mRNAs can use alternative translation initiation mechanisms to restore cellular homeostasis and respond to a variety of stresses, including those induced by toxins, oxidative insults, hypoxia, nutrient limitation, among many others. Each stress affects proteins that regulate cellular functions and impact protein synthesis [98,99].

## 2. The eIF2 Stress Response in NCDs 

### 2.1. The eIF2/ISR in Neurodegenerative Disorders

There is a poorly understood crosstalk of stress pathways that act on mRNA translation, particularly involving mTOR regulated eIF4E availability and the phosphorylation of eIF2α Ser-51 during the ISR, which is described here. During stress conditions, eukaryotic cells activate signaling pathways that induce the cellular ISR by carrying out eIF2α Ser-51 phosphorylation, which controls specific mRNA translation synthesis [100], as well as global protein synthesis [101]. To understand how translation is regulated by both the mTORC1 and eIF2 ISR signaling pathways under cellular stress, and their contribution to the development of NCDs, we first provide a brief overview of the eIF2 phosphorylation stress response and its impact on the mTORC1 signaling pathway.

A hallmark of many neurodegenerative diseases is the accumulation of misfolded proteins which is caused by the dysregulation of signaling pathways associated with ISR/UPR [102]. The phosphorylation of eIF2α Ser-51 by activation of any of its four kinases leads to inhibition of mRNA translation, as described earlier. In neurons, protein synthesis, most of which is cap-dependent, is required for synaptic plasticity and long-term memory formation, both of which are impaired during Alzheimer’s disease (AD) and Parkinson’s disease (PD) [102,103,104,105]. The biological hallmarks of AD are the accumulation of extracellular plaque composed of abnormally folded amyloid β (Aβ) protein, a cleavage product of amyloid precursor protein (APP), and the buildup of intracellular neurofibrillary tangles (NFT) of hyperphosphorylated protein tau [106]. Studies have shown abnormal hyper-phosphorylation of eIF2α in the hippocampus of APP/PS1 mice (a model of AD) and human AD patients [107,108]. To determine if elevated p-eIF2α contributes to the development of AD, researchers reduced levels of p-eIF2α by deleting PERK or GCN2 in APP/PS1 mice, allowing translation to occur, which improved synaptic plasticity [108]. Strikingly, APP/PS1 mice with either of these two eIF2α kinases deleted, showed restored levels of vital plasticity-related proteins and markedly improved synaptic and cognitive function comparable to that of wild-type (WT) mice [108]. Further studies also showed that suppression of PERK using a small molecule PERK antagonist, GSK2606414, prevented tau-mediated neuropathology, behavioral defects, and alleviated long-term depression (LTD), another synaptic plasticity impairment seen in AD [108,109,110]. The accumulation of the presynaptic neuronal protein α-synuclein (α-syn), can activate UPR signaling through PERK in dopaminergic neurons in the brain of PD patients [111,112,113], whereas GSK2606414 inhibition of PERK demonstrates neuroprotective capacity in a PD mouse model and prevents neuronal death in Parkin mutant flies [114,115]. Moreover, Guanabenz (GBZ), an inhibitor of GADD34 (a phosphatase that carries out eIF2α dephosphorylation), showed protection against stress-induced dopamine neurodegeneration in various PD models by enhancement of ATF4 and Parkin expression [116]. In addition, hypoxia and energy depletion have been recognized to inhibit the initiation of mRNA translation, leading to the accumulation of unfolded proteins in the ER, and to trigger the UPR likely as a means to restore protein balance in the post-ischemic brain [117,118]. Although the role of ER stress in brain ischemia remains unknown, it is possible that the activation of ER stress could cause neuron-protection, alleviating proteotoxic stress, as prolonged activation of ER stress aggravates ischemic injury [119]. Translation is also inhibited at the level of translation initiation during ischemia-reperfusion (IR) by a stress response involving p-eIF2α and activation of PERK [120,121]. Several studies have reported that after ischemia-reperfusion, there is an increase in mRNA and protein levels of GADD34, consistent with a short duration of p-eIF2α in brain reperfusion [122,123,124,125]. Notably, melatonin was reported to inhibit ER stress in neurons exposed to oxygen and glucose deprivation by decreasing p-PERK and p-eIF2α, thus reducing neuronal apoptosis and modulating protein levels in cerebral ischemia. These results indicate that attenuation of post-ischemic ER stress could be a potential therapeutic approach against neurodegenerative diseases [126].

### 2.2. The eIF2/ISR in Metabolic and Inflammatory Disorders

Metabolic disorders such as obesity, diabetes, liver cirrhosis, as well as cardiovascular diseases, are among the most severe risks to human health. Despite their different physiological and clinical symptoms, they share certain pathological characteristics, including intracellular stress and physiological organismal inflammation provoked by chronic metabolic stress, such as ER and oxidative stress [127]. P-eIF2α is essential to preserve ER integrity and to control insulin biosynthesis in β cells [128]. When this protective mechanism is compromised, it is thought to contribute to the onset of diabetes mellitus [128]. Analysis of mutations in PERK and eIF2α supports this view and suggests there could be a more complex role for the eIF2 stress response in regulating metabolism and diverse cellular functions of β cells [129]. For example, a mutation or knock-out (KO) of PERK demonstrated its essential requirement for the survival of pancreatic β cells and promoted early development of diabetes in mice [130,131]. A mutation in the phosphorylation site of eIF2α (eIF2α-Ser51Ala) when coupled to a high-fat diet in mice causes pancreatic β cell deficiency and induces symptoms of diabetes through induction of glucose intolerance and hyperglycemia [128,132]. Moreover, KO models of the other eIF2α kinases (HRI, GCN2, and PKR) did not develop significant defects in glucose homeostasis [133,134,135].

In an obese state, chronically elevated levels of free fatty acids and blood glucose can trigger ER stress, as well as insulin resistance, which can be abolished by increased production of insulin and other secreted proteins in the ER of pancreatic β cells [136]. It was shown that in hepatocyte-specific non-phosphorylatable (Ser51Ala) eIF2α knock-in mice, when fed a 60% high fructose diet, had an increase of liver fibrosis and hepatocyte death, suggesting that p-eIF2α could protect hepatocytes against oxidative stress [129]. Further, liver fibrosis involves a wound healing response leading to excessive accumulation of extracellular matrix (ECM) proteins and nodule formation [137]. Hepatic cirrhosis develops from liver fibrosis after a long period of liver-cell injury resulting from a necro-inflammatory response of continuous cell proliferation and inflammatory mediated cell death which causes chronic oxidative stress. Hyperammonemia, a high release of ammonia in the blood by the liver, is also associated with cirrhosis, as it alters protein homeostasis in the whole-body rather than just the liver [138]. However, hyperammonemia also induces a cellular stress response through p-eIF2α, which is mediated by GCN2, leading to translation inhibition by eIF2 phosphorylation due to reduced cellular amino acid pools [139]. This is consistent with the well-studied amino acid deficiency response, in which the ISR is activated by GCN2-eIF2α phosphorylation, increasing translation of ATF4 and GADD34 mRNAs. Importantly, during hyperammonemia, GCN2 is activated, but ATF4 and GADD34 are not translated, which results in dysregulation of both mRNA translation and proteostasis, the integrated ER-associated protein biosynthesis and degradation system [23,140]. Notably, nutritional deficits are also frequently detected in patients with cirrhosis, including low serum levels of metabolic substrates in plasma and muscle, resulting in skeletal muscle loss driven by hyperammonemia and reduced protein synthesis resulting from eIF2 phosphorylation [141,142]. Evidence that the phosphorylation of eIF2 is one driver of pathogenesis was shown by studies in which a single dose of a leucine enriched amino acid mixture in patients with alcoholic cirrhosis displayed a reduction in GCN2 activation. These results demonstrated that leucine deficiency results from molecular and metabolic stress in skeletal muscle and promotes pathogenic disease through the eIF2 phosphorylation pathway [140].

Oxidative stress often induces ER stress, as there is a tight crosstalk between the two stress responses, amplifying disease progression by induction of inflammation [127]. Consequently, oxidative stress induced by cigarette smoking exposure (CSE) causes accumulation of misfolded proteins by impairing ER folding capacity and increasing proteins involved in the endoplasmic-reticulum-associated protein degradation (ERAD) process, both proposed to play a role in the pathogenesis of COPD [143,144]. Upregulation of p-eIF2α is common in severe emphysema, as is ER stress in the lungs of COPD patients, and both cases are correlated with changes in levels of expression and phosphorylation of eIF2α and the severity of airflow obstruction [144]. During acute CSE in mouse models, dysregulation in ER homeostasis and induction of ER stress are observed in mice and involves phosphorylation of eIF2α by PERK. These results support the notion that pathogenesis caused by CSE results from an imbalance between proteostasis and apoptosis [145]. Gene expression profiling studies of bronchial airway epithelial cells have shown that in current and former smokers with and without COPD, that there is a significant enrichment of gene expression in the airway of COPD-associated alterations, mediated by ATF4 [146]. Blocking the end-stage activation of ATF4-mediated cell death is therefore desirable as a means of downregulating pathogenesis in airway disease. Accordingly, a selective inhibitor of eIF2α dephosphorylation, known as Salubrinal, maintains p-eIF2, inhibits ER stress-mediated apoptosis, and reduces overall protein synthesis [147]. Salubrinal protects against apoptosis in human bronchial epithelial cells after CSE, demonstrating that inhibition of protein synthesis through the eIF2 phosphorylation pathway is actually protective, and is a potential new approach for the treatment of COPD [146]. Salubrinal has also been used as a treatment to protect cardiomyocytes from apoptosis during heart failure (HF), the common end-stage of cardiac disease [147]. However, other studies that have utilized phosphor-mimetic mutants of eIF2α have shown oppositely that suppression of translation induces more severe HF phenotypes, and persistent p-eIF2α leads to cardiac insufficiency and HF development [146]. These different outcomes and results may not actually be opposed to each other, but rather, they show the need for dynamic regulation of eIF2α Ser51 phosphorylation in maintaining ER function. 

Under normal conditions, KO mice of the different eIF2α kinases (individually) display normal development and normal cardiac structure [133,148,149,150]. However, when subjected to transverse aortic constriction, GCN2 KO mice are less disposed to ventricular dysfunction, myocardial apoptosis, and fibrosis, compared to WT mice [149]. Likewise, PKR KO mice show preserved left ventricular contractility and reduced myocardial fibrosis when subjected to pressure overload, compared to WT mice; although the level of hypertrophy was similar between both groups [148]. By contrast, PERK KO mice showed impaired systolic function and increased myocardial fibrosis and apoptosis when subjected to transverse aortic constriction, compared to WT mice [148]. Normal development was also observed in HRI KO mice, except for mild macrocytosis (larger red blood cells) and hyperchromic (increased hemoglobin content) [133]. Further, GBZ treatment (an inhibitor of eIF2 Ser-51 phosphatase GADD34) protects against tunicamycin-induced ER stress in cardiac myocytes of rats, while mildly prolonging p-eIF2α [151]. Therefore, eIF2 phosphorylation serves a dual role as in other diseases and can be pathogenic in certain contexts, while protective in others.

**Figure 3:**
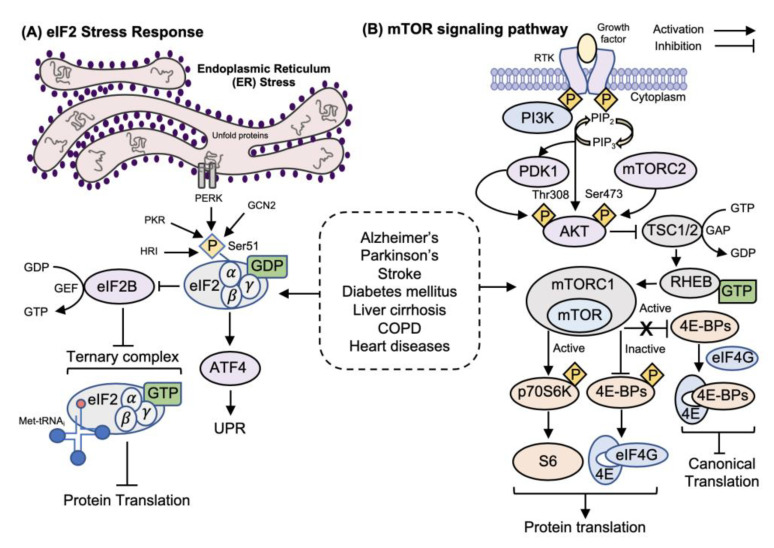
Human diseases linked to eIF2 Ser-51 phosphorylation stress responses and the mTORC1 signaling pathway. (**A**) Cellular stress can lead to the phosphorylation of eIF2 on Serine 51 of the α subunit (eIF2α Ser51) by four eIF2α kinases: heme-regulated inhibitor (HRI), protein kinase RNA- activated (PKR), PKR-like endoplasmic reticulum (ER) kinase (PERK), and general control nonderepressible 2 (GCN2); each responsive to different cellular stresses (schematic only showing ER Stress). Phosphorylation of eIF2α inhibits translation by blocking the activity of the guanine nucleotide exchange factor eIF2B, preventing the assembly of the eIF2–GTP–Met-tRNA_i_ ternary complex, and enhances translation of stress response mRNAs such as those encoding activating transcription factor 4 (ATF4), which can escape the inhibition of general translation by an indirect mechanism, resulting in the induction of downstream genes involved in the UPR. (**B**) In response to ligand stimulation (e.g., growth factors), cell receptors (e.g., RTKs) activate the kinase PI3K, phosphorylating PIP_2_ to produce PIP_3_, which then recruits AKT and allows phosphorylation of Thr308 and Ser473 through PDK1 and mTORC2, respectively. AKT inhibits the GTPase activity of TSC2 in the TSC1/TSC2 complex, elevating levels of GTP-Rheb, which subsequently enables activation of mTORC1. mTORC1 is then able to phosphorylate downstream proteins, p70S6K and 4E-BPs, resulting in regulation of mRNA translation. The phosphorylation and activation of p70S6K leads to activation of the ribosomal protein S6. Phosphorylation of the 4E-BPs results in 4E-BP inactivation and prevents their binding and sequestration of eIF4E, allowing interaction between eIF4E and eIF4G. When 4E-BPs are dephosphorylated, they become active, sequestering eIF4E, and blocking eIF4E/eIF4G interaction, resulting in eIF4E-mediated translation inhibition. Diseases linked to eIF2α phosphorylation and activation of mTORC1 signaling (described in the text) are Alzheimer’s, Parkinson’s, diabetes mellitus, cirrhosis, COPD, and heart diseases. ‘P’ in a yellow diamond indicates activating phosphorylation. eIF, eukaryotic translation initiation factor; GTP, guanosine triphosphate; tRNA, transfer ribonucleic acid; RTK, receptor tyrosine kinase; PI3K, phosphoinositide 3-kinase; PIP_2_, phosphatidylinositol (4,5)-bisphosphate; PIP_3_, phosphatidylinositol (3,4,5)-triphosphate; AKT, protein kinase B; PDK1, phosphoinositide-dependent kinase-1; mTORC, mammalian target of rapamycin complex; Rheb, RAS homolog enriched in brain; TSC, tuberous sclerosis; mTOR, mammalian target of rapamycin; p70S6K, 70-kDa ribosomal protein S6 kinase; 4E-BPs, eIF4E-binding proteins.

Additional studies in NCDs, are needed to more fully understand the mechanisms that lead to the activation of eIF2α kinases, subsequent events after eIF2α activation, and to better define the contrasting effects of eIF2 phosphorylation as disease pathogenesis mediating or disease protective. Although the inhibition of protein synthesis induced by p-eIF2α under stress can help alleviate protein misfolding and aggregation, the persistent activation of the UPR and prolonged inhibition of mRNA translation can also be deleterious for cells [106]. Furthermore, the phosphorylation of eIF2α is not the only mechanism that regulates mRNA translation initiation in many NCDs. As discussed next, inhibition of the mTORC1 signaling pathway can also downregulate mRNA translation by dephosphorylation of p70S6K and the 4E-BPs (Figure 3).

## 3. The mTORC1 Signaling Pathway in NCDs

### 3.1. The mTORC1 Signaling Pathway in Neurodegenerative Disorders

Many studies have demonstrated a key role for the mTORC1 pathway in pathological responses in neuronal function and development. Activation of mTORC1 signaling is crucial for long-lasting forms of synaptic plasticity in learning and memory [152]. The brains of patients with advanced Alzheimer’s disease (AD) showed a systematic disorder of protein synthesis that is dominated by increased levels of phosphorylated (p) proteins, including p-mTOR (hyperactivated), p-p70S6K, p-4E-BP1 (inactivated), and p-eIF4E (likely involved in the increased activity) [153,154,155]. A positive correlation between total tau and p-tau expression has also been shown, suggesting that increased activity of the mTORC1 signaling pathway may contribute to increased levels of tau, possibly its hyperphosphorylation and its increased accumulation in NFT-bearing neurons [153,154,155]. Rapamycin is a well-known mTOR allosteric inhibitor that has been used for decades to suppress T cell responses. Rapamycin and its analogs have shown some potential as an anti-AD drug by ameliorating Aβ and tau pathologies [156]. When mTORC1 is inhibited in rat neural cells and human neuroblastoma cells, the APP mRNA switches from cap eIF4E/cap-dependent translation to translation from its intrinsic 5′UTR IRES. These results suggest that IRES-mediated translation is important for the synthesis of APP and development of the disease, and provides a possible approach to block the progression of AD by impairing the translation activity of the APP IRES [157]. However, reducing the mTORC1 pathway by the administration of rapamycin is not likely a good approach, as it could impair development and proper synaptic plasticity for learning and memory, and cortical development in AD, in addition to its immune-suppressing effects [158]. Further evidence for the increased activity of the mTOR pathway in development AD comes from studies with ATP-competitive mTOR inhibitors that block mTOR directly. These catalytic mTOR inhibitors have been shown to rescue or correct hyperactivated mTOR signaling by induction of autophagy, and have demonstrated effectiveness at blocking the phosphorylation of 4E-BP1, showing reduced hallmarks of AD, and recovered cognitive performances [159,160,161,162]. 

In Parkinson’s disease (PD), expression levels of mTOR were found to be significantly increased at the mRNA and protein levels in the temporal cortex of patients with clinical dementia, particularly in neurons displaying accumulation of α-syn [163]. Mutations in PD patient-derived fibroblasts cause a reduction of p-S6K levels, leading to decreased lysosomal recycling, which is essential for the autophagic clearance of α-syn [164], again consistent with the role of hyperactive or continuously active mTORC1 in PD development and/or disease progression. Moreover, leucine-rich repeat kinase 2 (LRRK2), is a large multidomain protein with GTPase and kinase activity that phosphorylates and modulates 4E-BP1/eIF4E interaction [165]. There are many LRRK2 mutations reported in PD patients, of which the G2019S mutation in the kinase domain is the most prevalent, and has been shown to regulate the mTOR pathway via p-4E-BP1 and AKT, resulting in an increase in bulk translation, which leads to neurotoxicity and neurological disorders [166,167]. In addition to LRRK2 mutations, eIF4GI has also been associated with PD mutations, where it is proposed to have a role in the synthesis of mitochondrial proteins involved in PD pathogenesis [168,169]. However, a precise role for eIF4GI needs greater characterization, as it remains unclear how it is involved with other PD predisposing genes and effects. Interestingly, rapamycin along with an S6K inhibitor (PF-4708671) was able to revert the cognitive and affected symptoms of PD in mice, including depression and bipolar disorder, and rapamycin administration during several stress conditions stimulates α-syn mRNA translation via its 5′-UTR IRES element [170,171]. Given the potential role of mTORC1 in neurological diseases, a few studies have confirmed that after brain ischemia, the decrease in oxygen (by blockage), glucose and growth factors, triggers a reduction in mTORC1 activity [172]. Accordingly, the phosphorylation and activation of mTORC1 was shown to improve memory function and provide neuroprotection during remote ischemic pre-conditioning (induced before stoke) [173]. During ischemic post-conditioning (blood reperfusion after stroke), long-term brain focal ischemic damage and neurological disability are decreased with increased mTORC1 activity, mediated by increased activities of mTOR, S6K, and p-4EBP1 [174]. Other studies have shown that mTOR could provide neuroprotection against cerebral ischemic insult and irradiation injury, all by inhibition of apoptosis and autophagic cell death in neurons [175,176,177].

### 3.2. The mTORC1 Signaling Pathway in Metabolic and Inflammatory Disorders

The mTORC1 pathway is involved in mRNA translation effects that are disrupted in certain metabolic and inflammatory diseases. mTOR activity in normal cells is involved in insulin resistance, muscle oxidative metabolism, white adipose tissue differentiation, and β cell-dependent insulin secretion [178,179,180]. Notably, dysregulation of the mTORC1 pathway has been implicated in the development of several diseases that involve metabolic and inflammatory changes, such as obesity, which can lead to diabetes, pulmonary and cardiovascular disorders such as stroke and others [180]. S6K1 inhibition has been suggested as a new therapeutic target to improve glucose disposal in obese patients, with one study demonstrating that S6K KO mice fed with a high-fat diet (HFD) were protected against diet-induced obesity, whereas S6K KO mice were still insulin sensitive [181]. Like S6K1, the 4E-BP translational repressors have been found to play a critical role in body weight and glucose homeostasis [182,183,184]. One study reported that high levels of glucose and insulin-stimulated phosphorylation of 4E-BP1 and activation of mTOR, promoting the release of sequestered eIF4E in type 2 diabetes [184]. Double deletion of the two major 4E-BPs (1 and 2) in mice promotes the development of obesity through reduced energy expenditure, lipolysis, increased adipogenesis, and insulin resistance [182]. While restoration of 4E-BP1 expression in 4E-BP KO mice protects them against HFD-induced obesity and insulin resistance, intriguingly, this effect was only observed in male mice [183]. Interestingly, two new studies in mice showed that increased translation of two proteins in the liver, TET3 and HNF4a, results in increased production of blood glucose and insulin. Specifically, it was shown that abnormal protein signaling of TET3 contributes to the development of fibrosis in the liver [185,186]. Based on these data it has been proposed that targeting the TET3 and HNF4a proteins could reverse type-2 diabetes and liver fibrosis. In addition to fibrosis, patients with cirrhosis can also develop a recognized complication, known as skeletal muscle wasting or sarcopenia, due to an imbalance between protein synthesis and degradation of skeletal muscle proteins resulting from chronic alcohol abuse [187,188,189]. It was reported that in chronic alcohol-fed rats a decrease in the translation of skeletal muscle protein mRNAs results from a reduction in p-mTOR and p-S6K levels, increased in 4E-BP1/eIF4E association, and reduced levels of active eIF4E/eIF4G complex [190]. 

When respiratory muscles are impacted by COPD, skeletal muscle wasting or cachexia often develops, in which insulin-like growth factor 1 (IGF-1) signaling plays a major role in promoting disease onset and progression, resulting from the activation of the mTOR signaling pathway [191,192,193,194]. The severity of COPD has been linked to significantly low levels of IGF-1 in serum, with cachectic COPD patients showing decreased protein levels of IGF-1 in muscle [195,196,197,198]. The activation of mTOR in mice induces lung cell senescence, lung emphysema, pulmonary hypertension, inflammation, and lung alterations resembling those in COPD [199]. COPD patients with low muscle mass exhibit an increase in phosphorylation of the downstream targets 4E-BP1 and p70S6K, explained by an attempt to compensate for the loss of muscle mass through increased protein synthesis compared to patients with normal muscle mass [193,200]. The relationship between COPD and mTOR activation has been identified as a new therapeutic target in COPD [201]. Furthermore, it has been reported that the 5′-UTR of the IGF-1 receptor mRNA is translated by IRES-dependent translation, providing additional control for its expression under stress conditions that downregulate eIF4E/mTORC1-dependent canonical mRNA translation [202].

Lastly, it was shown that reduction of mTOR signaling in adult mice induces the development of heart failure due to the accumulation of dephosphorylated 4E-BP1. In fact, when both mTOR and 4E-BP1 are deleted, heart failure phenotypes are improved due to unrestricted availability of eIF4E [203]. Another study reported that canonical translation initiation is strongly inhibited following myocardial infarction, through a mechanism involving sequestration of eIF4E by 4E-BP1, although several other forms of regulation, including inhibition of Rheb/mTORC1 signaling were also observed, suggesting several paths are involved to 4E-BP control [204,205,206]. In ischemic heart diseases, inhibition of mTOR attenuates adverse myocardial remodeling and improves cardiac function by blocking canonical translation through 4E-BP1 [207]. In fact, within 10 minutes of ischemia, 4E-BP1 is activated, negatively regulating translation, but then shifts to an inactive state, inducing a global activation of translation in the non-infarcted muscle to compensate for cardiomyocyte loss [205]. Although canonical eIF4E-dependent translation is downregulated, some mRNAs are still translated in the hypoxic environment, which likely involves a switch to eIF4E-independent translation via an IRES is or the DAP5/eIF3d complex [208,209,210]. Therefore, non-eIF4E-mediated mRNA translation plays a major role during ischemia, as most lymphangiogenic factors are translationally induced in hypoxic cardiomyocytes [208,209,210,211]. Finally, consistent with these findings, inhibition of mTORC1 by rapamycin was recently shown to be cardioprotective in pressure-overloaded and ischemic heart diseases, preventing cardiomyocyte apoptosis, and promoting autophagy in chronic heart failure [212].

## 4. Conclusions and Future Directions

Translation regulation is essential in maintaining cell division, survival, as well as protein and cellular homeostasis [213,214,215,216,217,218]. Studies of NCDs that result from dysregulation in protein synthesis, specifically the rate-limiting step of mRNA translation initiation, provide a new understanding of the different key mechanisms of mRNA translation in the pathogenesis of distinct diseases. As described in this review, translation is mediated by eukaryotic initiation factors (eIFs), which have different functions in controlling the rate of initiation and regulate gene expression during diseases. Mechanisms that drive selective translation of specific mRNAs, such as eIF4E/mTORC1-dependent, DAP5/eIF3d-dependent, and IRES-dependent mRNA translation demonstrate plasticity in the use of different translation initiation mechanisms and a range of requirements for the translation factors involved. A greater characterization of these mechanisms will provide a critical understanding of their roles in the pathogenesis of NCDs. Under stress conditions, specific proteins are altered during translation, which may lead to a predisposition to several comorbid diseases described here, such as Alzheimer’s disease, Parkinson’s disease, stroke, diabetes mellitus, cirrhosis, COPD, and heart diseases. The majority of cap-dependent mRNAs are regulated at the level of initiation by the mTORC1 signaling pathway through control of cap binding protein eIF4E, which is important for cell proliferation, regulation of cell survival, and inflammatory responses among many others. During a number of stress conditions (e.g., ER stress, amino acid starvation, hypoxia, etc.), IRES-mediated or non-canonical cap-dependent translation initiation, including DAP5/eIF3d directed translation initiation ensues, allowing cells to adapt to a new state, by translating specific stress response and adaptation mRNAs including those encoding survival proteins. Activation of eIF2 stress kinases and inactivation of the mTORC1 pathway are central to these adaptive mechanisms. It needs to be noted that the mTOR and eIF2 phosphorylation pathways also regulate the rate of translation elongation, which impacts on protein synthesis rates and even the types of proteins made. This review however focused on the control of translation initiation of both pathways in human non-communicable diseases. 

The phosphorylation of eIF2α was shown to be critical in the diseases discussed, leading to repression of long-term memory formation and synaptic plasticity in AD [104,105], and protein aggregation and dopaminergic neurodegeneration in PD [111,112,113]. Pathogenic eIF2α phosphorylation is also involved in the dysregulation of protein balance in the post-ischemic brain [117,118]. In endocrine organ diseases, aberrant metabolism, function and survival of pancreatic β cells for example, as well as the development of glucose intolerance, hyperglycemia, and diabetes all involve pathogenic complications of the eIF2α phosphorylation pathway [128,129,130,131,132]. This is also observed as a driver of liver fibrosis, hepatocyte death, and skeletal muscle loss driven by hyperammonemia during hepatic cirrhosis [141,142,219]. Severe emphysema and airflow obstruction in the lungs of COPD patients, as well as ventricular dysfunction, apoptosis, and fibrosis of the myocardium, cardiac insufficiency, and heart failure, are driven in part by pathogenic mechanisms of eIF2α phosphorylation [144,148,149,220,221]. These findings highlight the critical role played by organ, tissue, and cellular physiological stress responses through eIF2α phosphorylation, and highlight eIF2α kinases as potential therapeutic targets. The other primary mechanism regulating translation in NCDs discussed in this review is the mTORC1/eIF4E regulation pathway, contributing to increased hyperphosphorylated tau protein accumulation in NFT-bearing neurons during AD [153,154,155], and neurotoxicity and accumulation and spread of pathogenic α-syn in PD brains [164,165,166,167,222]. Neuroprotection and improved memory function during remote ischemic pre-conditioning, decreased long-term brain focal ischemic damage, and neurological disability during ischemic post-conditioning [173,174], which also involve pathogenic mTORC1/eIF4E signaling and regulation. Similarly, it is well established that insulin resistance and co-morbid inability to properly regulate body weight and glucose homeostasis in diabetes involves the mTORC1/eIF4E pathway [178,179,182,183,184]. Skeletal muscle hyperammonemia of liver cirrhosis [142,190], lung emphysema, pulmonary hypertension and inflammation, lung alterations in COPD, and development of a variety of heart failure clinical manifestations including acute myocardial infarction all involve pathogenic mTORC1 signaling and eIF4E regulation [199,203,204,205,206]. The studies described here provide evidence for the often collective roles of these two pathways in controlling translation and promoting disease when disrupted as a result of physiological stresses and biological disease mediators. 

Given the complexity of NCDs and the plasticity of translational regulation, more research is needed to better understand how pathogenic mechanisms arising from these different pathways develop and promote the progression of NCDs. Future studies are needed to address whether differential sensitivity to physiological and biological activators of stress responses or the cell/tissue-intrinsic differences in translation regulation, as well as mutations in UTRs, play causal roles in the development of NCDs. As noted, there are major points of commonality among the different NCDs described here, involving translational regulation by eIF2 and mTORC1/eIF4E stress response pathways. A better understanding holds great potential of being useful in the development of diagnostic and prognostic tools as well as new therapeutics.

## Abbreviations

NCDs	Non-communicable diseases
eIF2	eukaryotic initiation factor 2
mTORC	mammalian target of rapamycin complex
4E-BPs	eukaryotic initiation factor 4E- binding proteins
AD	Alzheimer’s disease
PD	Parkinson’s disease
COPD	Chronic obstructive pulmonary disease
